# Severe Complications Due to Biopolymers in a Patient With Autoimmune Disease: A Case Report and Review of the Literature

**DOI:** 10.1093/asjof/ojaf099

**Published:** 2025-08-04

**Authors:** Andrés Hormaza-Jaramillo, Daniela Peñaloza Gonzalez, Sara Alejandra Benavides-Ibarra, Natalia Calvache Hernandez, Liliana Eugenia Muñoz Garcia, Alejandra Hidalgo Cardona, Jackeline Murrillo-Mera

## Abstract

Biopolymers are frequently used for aesthetic or reconstructive purposes, often without adequate consideration of the potential long-term health complications they may cause. The use of these substances has been associated with autoimmune/autoinflammatory syndrome induced by adjuvants (ASIA syndrome); however, the existing literature is limited, and there is currently no clear consensus regarding its management. This case report aims to describe a case of severe biopolymer-induced inflammatory disease in a patient with a diagnosis of rheumatoid arthritis, highlighting both the diagnostic and therapeutic approaches. This case report examines a 58-year-old female patient with a history of biopolymer application to the gluteal and facial region 20 years before and a diagnosis of rheumatoid arthritis for the past 2 years. She presented with severe inflammatory skin lesions with worsening arthralgia. An MRI scan revealed exogenous material with signs of severe inflammation of the surrounding tissues, and a biopsy was consistent with foreign body granulomas with a “Swiss cheese” morphology. The patient was hospitalized and received multidisciplinary management, achieving an excellent clinical response. This case highlights the importance of a comprehensive evaluation and a multidisciplinary approach in patients with autoimmune disease and previous exposure to biopolymers. The coexistence of autoimmune diseases can aggravate the inflammatory response, suggesting the need for additional studies exploring this interaction and its prognostic impact.

**Level of Evidence: 5 (Risk)**  
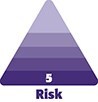

For more than 3 centuries, injections of various exogenous substances have been used for aesthetic or reconstructive purposes, under the premise of being simple, painless, inexpensive, and apparently safe methods.^1,2^ However, it is now known that these substances can cause irreversible damage to the body, posing a public health problem, especially in Latin American countries where their use is more common.^3^

Among these substances are biopolymers, macromolecules of natural or synthetic origin, used as tissue fillers. Silicone derivatives, such as polyvinyl methacrylate and polymethyl siloxane, are the most commonly used for aesthetic purposes.^4,5^ These substances can trigger a severe local inflammatory reaction, which may present acutely or delayed. Furthermore, migration to other areas of the body has been reported, compromising vital organs and putting the patient's life at risk.^5^

Various terms have been used to describe the clinical manifestations caused by the injection of these substances.^3^ In 2011, Shoenfeld and Agmon-Levin^19^ proposed the term autoimmune/autoinflammatory syndrome induced by adjuvants (ASIAs) to characterize the clinical manifestations resulting from an immune response to adjuvants, which may or may not fulfill established classification criteria for certain immune-mediated diseases—such as lupus, rheumatoid arthritis, inflammatory myopathies, systemic sclerosis, vasculitis, or sarcoidosis—or even fibromyalgia. This syndrome is considered a diagnosis of exclusion, in which clinical evaluation and personal history play a fundamental role.^6^

Despite advances in our understanding of this pathology, there is still no consensus on the proper management of this condition and its potential complications, making it difficult to diagnose and treat, with an uncertain prognosis, representing a challenge for the clinician.^2,7^ Therefore, it is important to report these types of cases. Below, we present the case of a patient with immune-associated disease associated with biopolymers, who was presented with severe inflammation with multiple cutaneous and systemic manifestations. She received multidisciplinary management with evident clinical improvement.

## CASE PRESENTATION

A 58-year-old female patient with a history of hypothyroidism and high blood pressure. She had applied a contouring substance to her face and buttocks ∼20 years ago; the material used is unknown. Breast augmentation surgery was performed 14 years ago. Two years ago, she was diagnosed with rheumatoid arthritis, seropositive for rheumatoid factor (40 IU/mL), positive for anti-CCP (94 IU/mL), and hypogammaglobulinemia (without clinical repercussions). Initially treated with leflunomide 20 mg daily and adalimumab 40 mg subcutaneously every 2 weeks, she was subsequently treated solely with Deflazacort 6 mg daily. She had no significant family or psychosocial history.

She previously consulted another institution for a 3-month history of painful, erythematous nodules on her face, associated with eyelid and facial edema. She also presented with similar lesions on her buttocks, involuntary weight loss, and night sweats. Malignancy was ruled out, and the patient reported being treated with antibiotics and analgesics, which she cannot recall.

**Figure 1.**
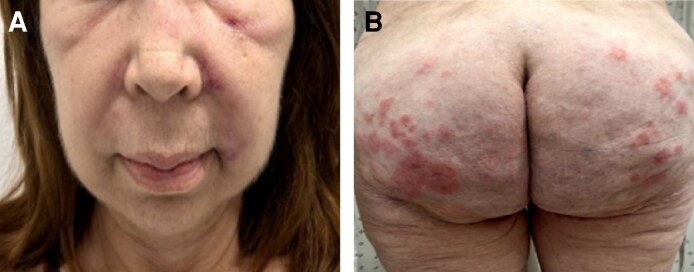
Images of a 58-year-old female patient with injuries to the (A) face and (B) buttocks upon admission.

She consulted our institution with a 1-day history of mild dyspnea and moderate joint pain in the hands, without edema. This was associated with worsening skin lesions on the face and buttocks. Upon admission, the patient's vital signs were as follows: blood pressure: 150/79 mm Hg, heart rate: 67 LPM, SO_2_: 99%, respiratory rate: 18 RPM. Physical examination revealed a patient with acceptable general condition. Dermatological examination revealed a Phototype III patient with marked facial and bilateral eyelid edema. The patient's forehead presented erythematous subcutaneous nodules, some with a yellow crust in the center, a cobblestone appearance, indurated, infiltrated, not adherent to deeper planes, and painful to palpation. The patient also presented tissue thickening at the glabellar level. The buttocks showed erythematous plaques and nodules, painful to palpation (Figure 1). The oral cavity also showed mild subglottic edema.

She was referred to the emergency department, where treatment was initiated with hydrocortisone 50 mg every 8 h, colchicine 0.5 mg every 12 h, and hydroxyzine 25 mg every 24 h. Because of the suspicion of a granulomatous reaction or a superinfected foreign body reaction, the decision was made to admit her for multidisciplinary management.

Acting clinical tests showed a complete blood count within normal ranges, preserved renal function, elevated aspartate aminotransferase (AST) (64 U/L), alanine aminotransferase (ALT) 58 U/L, and lactate dehydrogenase (LDH; 284 U/L). Immunological tests showed rheumatoid factor (40 IU/mL), anti-CCP antibodies (94 IU/mL), and positive antinuclear antibody (ANA; 1:640 dilutions with homogeneous AC-1 pattern), with no other laboratory abnormalities (Tables 1, 2). Multiple paraclinical tests ruled out a possible infectious etiology (Table 3).

**Table 1.** Laboratory Tests

Test	Initial results	1-week follow-up result
Hematology		
White blood cells	6.38 × 10^6^ μL	14.46 × 10^6^ μL
Neutrophil	5.02 × 10^3^ μL	Not reported
Lymphocytes		Not reported
Eosinophils	0 × 10^3^ μL	Not reported
Hemoglobin	11.8 g/dL	Not reported
Hematocrit	37 g/dL	Not reported
Platelet	239 × 10^3^ μL	274 × 10^3^ μL
Biochemistry		
Thyroid-stimulating hormone	1.140 μUI/mL	Not reported
AST	64 U/L	87 U/L
ALT	58 U/L	72 U/L
Protein C	0.06 mg/dL	0.19 mg/dL
Lactate dehydrogenase	284 U/L	Not reported
Erythrocyte sedimentation rate	15 mm/h	24 mm/h
Angiotensin-converting enzyme	26.6 U/dL	Not reported
Folate	11.2 ng/mL	Not reported
Ferritin	56.5 ng/dL	Not reported
Creatinine	0.76 mg/dL	0.74 mg/dL
Ureic nitrogen	17.3 mg/dL	Not reported
Potassium	3.98 mmol/L	Not reported
Sodium	143.2 mmol/L	Not reported

**Table 2.** Autoimmune Tests

Immunology	
Test	Result
Antinuclear antibodies—indirect immunofluorescence	1:640 dilutions homogeneous patter AC-1
Proteinase 3 antibodies	1.5 U/mL
Myeloperoxidase antibody	0.8 U/mL
Anti-DNA manual	17.9 IU/mL
Immunoglobulin E	2.9 g/L
Immunoglobulin G	11.25 g/L
Rheumatoid factor	40 UI/mL
Cryoglobulins	Negative
Protein electrophoresis	Normal electrophoresis pattern

**Table 3.** Infectious Tests

Infectious diseases	
Test	Result
Mycobacteria culture on skin	Negative
Bacilloscopy staining	Negative
Aerobic bacteria culture on skin	Negative
Deep mycosis fungi culture on skin	Negative
KOH microscopic examination on skin	Negative
Gram staining	Negative
Hepatitis A antibodies	3.860 S/CO
Hepatitis B surface antigen (Ag HBs)	0.33
Hepatitis B surface antibodies (anti-HBs)	<2 UI/L
Hepatitis B total core antibodies (Anti-Core-HBc)	2.080
Hepatitis C antibodies	0.070 S/CO
Treponema pallidum antibodies (Treponemal test)	0.05 S/CO
HTLV I and II total antibodies	0.16 S/CO
HIV I and II antibodies	0.19
Cryptococcus antigen	Negative
Histoplasma capsulatum urinary antigen	Negative

HIV, human immunodeficiency virus; HTLV, human T-lymphotropic virus 1.

**Figure 2.**
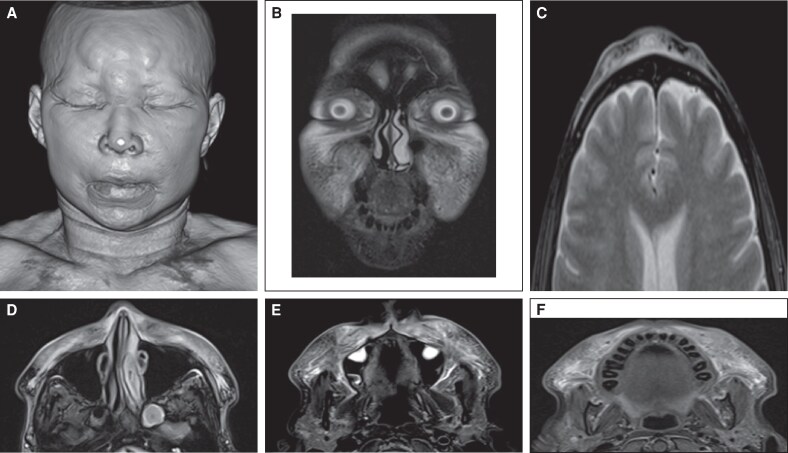
MRI of the paranasal sinuses and facial region using a T_2_-weighted fat-suppressed sequence in coronal and axial sections. In a 58-year-old woman with a history of nonabsorbable exogenous substance injections, diffuse edema of the skin and subcutaneous tissue is observed without fluid collections in the following areas: (A) volumetric facial reconstruction; (B) facial region; (C) frontal fat; (D) nasal and zygomatic regions, with mucosal opacification containing high-protein material in the left sphenoid sinus; (E) nasolabial region; and (F) perioral space, maxillary fat, and retromaxillary fat.

**Figure 3.**
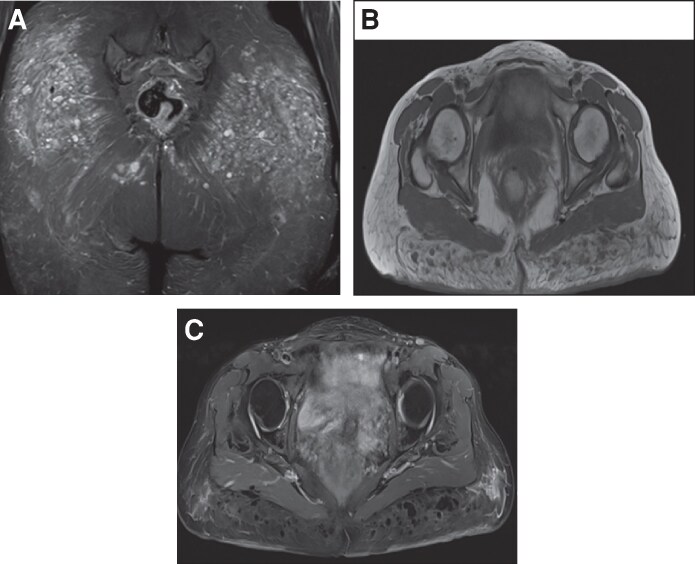
Pelvic MRI of a 58-year-old female patient. (A) Coronal slice, Short TI inversion Recovery (STIR) sequence. (B) axial slice, T_1_-weighted sequence. (C) axial slice, proton density sequence. Multiple nodular images are observed, hyperintense on the STIR sequence (A) and hypointense on the T_1_-weighted and proton density sequences (B and C), with a maximum diameter of 1 cm. These lesions are randomly distributed throughout the subcutaneous tissue, fascia, and some superficial muscle fibers of the right gluteus maximus muscle. Based on the clinical history, they correspond to nonresorbable exogenous material.

Contrast-enhanced MRIs of the face and paranasal sinuses revealed the presence of foreign material, with signs of severe inflammation in the surrounding tissues. The remaining findings are described in Figures 2 and 3.

**Figure 4.**
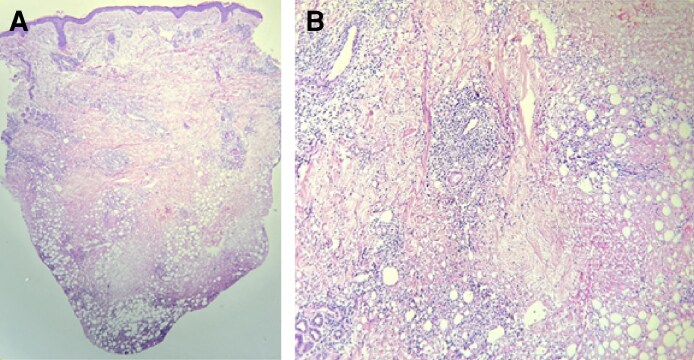
Histopathological analysis of skin biopsies from (A) the left forehead and (B) the right gluteal region in a patient with a 30-year history of biopolymer injection. Macroscopically, 2 small skin fragments were received: 1 from the left forehead (0.4 × 0.3 cm) and 1 from the right gluteal area (0.6 × 0.5 cm). Microscopically, both samples showed epidermis with spongiotic changes. The dermis revealed disintegration of adipose tissue, with multiple variably sized vacuolated spaces surrounded by a mixed inflammatory infiltrate consisting of lymphocytes, plasma cells, histiocytes, neutrophils, and foreign-body giant cells. Inflammation extended into the superficial and deep dermis, as well as into the interstitial and subcutaneous fibrous tissue. The overall appearance resembled a Swiss cheese pattern. Additionally, nuclear-dusted neutrophils and red blood cell extravasation were noted. Special stains (Gram, PAS, and Ziehl–Neelsen) were negative for microorganisms. Findings were consistent with a foreign body reaction to exogenous material.

Two skin biopsies were taken from lesions located on the frontal region of the face and the right buttock. The results showed dermis with adipose tissue disaggregation, revealing spaces of varying sizes surrounded by a mixed inflammatory reaction, with lymphocytes, plasma cells, histiocytes, neutrophils, and giant cells to a foreign body, distributed periadnexally, superficially and deeply perivascularly, and interstitially down to the subcutaneous tissue. In addition, fibrous tissue was present, with a general “Swiss cheese” appearance (Figure 4). Gram, PAS, and BK stains were negative for microorganisms.

These findings confirmed the diagnosis of a foreign body reaction (exogenous material), and infectious etiologies were ruled out. Treatment was initiated with prednisone 25 mg daily. Subsequently, her clinical condition improved, and laboratory results were within normal ranges, leading to her discharge after 6 days of hospitalization. She was continued on methotrexate 2.5 mg twice weekly, colchicine 0.5 mg every 12 h, prednisolone 5 mg daily, and folic acid 5 mg once weekly.

Additionally, during follow-up, the patient was found to have elevated transaminase levels. Although the values were not markedly high (AST: 87 U/L; ALT: 72 U/L; Table 1), they were interpreted as a possible early manifestation of methotrexate-associated hepatotoxicity. Given the concomitant use of colchicine and corticosteroids, along with an active systemic inflammatory state, a preemptive dose reduction was implemented in accordance with international recommendations regarding hepatotoxicity. This decision was accompanied by close clinical monitoring and outpatient follow-up, with subsequent normalization of liver function tests.

**Figure 5.**
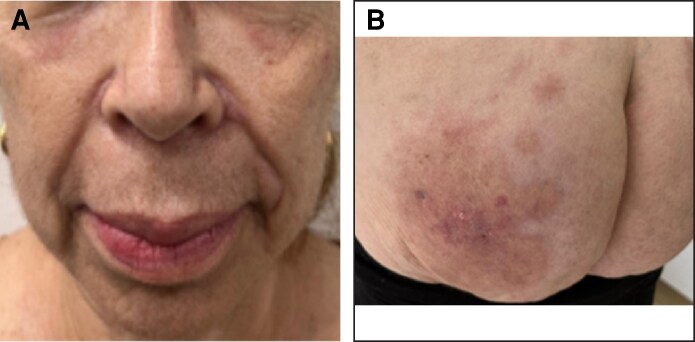
Images of a 58-year-old female patient's (A) facial and (B) gluteal lesions after treatment.

At the follow-up appointment with rheumatology 1 month after discharge, the patient's clinical progress was excellent, with clear improvement in the facial and gluteal skin lesions, and a marked decrease in the signs of inflammation and associated edema (Figure 5). There were no paraclinical changes. The patient is currently clinically stable with the prescribed pharmacological treatment, awaiting biopolymer removal surgery.

## DISCUSSION

Biopolymers are frequently injected for aesthetic or reconstructive purposes, as in the present case.^1,2^ A 58-year-old female patient with a history of autoimmune disease and application of contouring agents to her face and buttocks presented a case. After 20 years of application of this agent, she began to present severe inflammatory skin lesions associated with joint pain.

It is known that biopolymer agents, when they encounter the recipient tissue, can trigger an excessive local inflammatory reaction, proportional to the molecular weight of the infiltrated agent.^5^ In many cases, the patient is unaware of the material and the actual amount infiltrated, as is the case in this case.^8^ The mechanisms and clinical presentation of this disease may differ depending on the injected material, which is one of the major obstacles to its recognition.^1^

The injection of these products can cause different clinical manifestations, which can be local or systemic. Depending on their time of onset, they can be classified as immediate or delayed and can include a wide variety of symptoms, ranging from local manifestations to systemic complications, including migration of the injected material.^5^ In the present case, the patient presented with a late-onset clinical picture (20 years after the injection), with local clinical manifestations that included the appearance of multiple erythematous and painful nodules associated with edema, as well as systemic clinical manifestations, including marked joint pain.

The signs and symptoms associated with biopolymer injection have received various names, initially described as “iatrogenic allogenesis” by Coiffman.^15^ Later, terms such as “allogenic disease” and “biopolymer diseases” were introduced.^3^ In 2011, Shoenfeld and Agmon-Levin proposed the term “adjuvant-induced autoimmune/autoinflammatory syndrome” to describe the clinical manifestations arising from the immune response to adjuvants, substances that enhance the immunogenicity of an antigen without triggering a direct immune response.^6^ They also established diagnostic criteria, later modified by Alijotas-Reig in 2015 for greater objectivity.^9^

ASIA syndrome is a diagnosis of exclusion, where clinical features, medical history, and certain antibodies are keys.^6^ In our patient, a history of filler application to the face and buttocks was very useful for the diagnostic approach. In this case, the Alijotas-Reig criteria were considered for diagnosis. These 12 clinical criteria are used, and the diagnosis is made in the presence of 2 major criteria or 1 major and 2 minor criteria.^6,10^ Our patient met 4 major criteria: previous exposure to biomaterials, latency greater than 1 month, clinical involvement, and biopsy findings, and a minor criterion: the presence of autoantibodies and elevated lactate dehydrogenase (Table 4).

**Table 4.** Autoimmune/Autoinflammatory Syndrome Induced by Adjuvants Syndrome Criteria

Criteria	Case report
Major criteria	
1. Exposure to external stimuli: biomaterials,^a^ vaccines, anilines, or other organic/inorganic materials before the appearance of clinical manifestations	Yes
2. Minimum latency period of days in the case of vaccines and 1 month when the trigger is suspected to be other than vaccines, that is, biomaterials^b^	Yes
3. Clinical involvement: Local/regional: inflammatory skin nodules, edema, or angioedema. Skin indurations: pseudoabscesses; lymphadenopathy, panniculitis, morphea, and sarcoid-like lesions Systemic: distant inflammatory nodules, arthritis, sicca or Sjögren's syndrome,^c^ myositis,^d^ muscle weakness,^e^ widespread panniculitis, demyelinating neurological involvement Evolution to autoimmune disease with or without organ involvement	Yes
4. Foreign body biopsy of the affected area or lymph nodes^f^ or histological findings consistent with autoimmune/granulomatous disorders	Yes
5. Withdrawal of triggering materials induces improvement	NA
6. Presence of compatible HLA (eg, HLA B8, HLA DRB1, HLA DR3, HLA DQB1, or a combination of haplotypes)	NA
Minor criteria	
1. Recent history of triggering factors before the onset of clinical manifestations^g^	No
2. Large de novo livedo reticularis and/or erythema on the hands that appears at the onset of clinical manifestations^h^	No
3. Presence of autoantibodies and/or hypergammaglobulinemia and/or elevated ACE and/or elevated LDH and/or low complement levels	Yes

Modified from Alijotas-Reig.^9,10^ ACE, angiotensin-converting enzyme; HLA, human leukocyte antigen; LDH, lactate dehydrogenase; LES, systemic lupus erythematosus; MRC, Medical Research Council muscle strength grading system; NA, not applicable. ^a^Paraffin, silicone, medical-grade silicone, methacrylate, poly-L-lactic acid, polyacrylamide, polyalkylimide, collagen, hydroxyapatite, hyaluronic acid, stabilized hyaluronic acid of nonanimal origin, alginate. ^b^When adverse reactions occur after a second challenge, a short period of time may be acceptable. ^c^Demonstrated through objective testing: salivary flow or salivary scintigraphy; Schirmer and Rose Bengal tests. ^d^Documented through elevated muscle enzymes and/or electromyography and/or muscle biopsy. ^e^Muscle strength should be assessed using the MRC scale or similar. ^f^Specifying the type of histopathological pattern identified (eg, sarcoid, palisade, necrobiotic, paraffinoma, and siliconoma). Specific histopathological patterns of biomaterials and vaccine adjuvants should be considered and described. ^g^Infectious process, trauma, or manipulation of filled/implanted/vaccinated areas. ^h^Appearance of painless, symmetrical, and bilateral de novo erythema on the palms and sometimes on the palmar surface of the fingers, with a reddish, purplish, or bluish color, like that seen in LES, hyperestrogenism, or vasculitis.

Although the case was suggestive of ASIA syndrome, it was important to rule out infectious etiology. Therefore, paraclinical tests were performed, which ruled out infection (Table 3). Additionally, paraclinical tests were performed to evaluate the patient's systemic involvement. An elevated LDH (284 U/L) and positive ANA (1:640 dilutions with a homogeneous AC-1 pattern) were found (Tables 1, 2). Elevated acute-phase reactants and polyclonal hypergammaglobulinemia are common initial findings in ASIA syndrome, although these may not be significantly elevated. Furthermore, the identification of specific antibodies associated with the syndrome can be very useful.^6,10^ In our patient, these results supported the diagnostic suspicion (Table 2).

Different types of imaging studies can be performed, depending on the specific characteristics of each patient.^10^ Contrast-enhanced MRI has proven useful for localizing allogeneic substances, both localized and distant, as well as for documenting granulomas in subcutaneous tissue and muscles.^6^ For this reason and considering that the patient had lesions in the buttocks, a pelvic MRI study was performed. This study reported multiple images suggesting the presence of biopolymers and ruled out migration of the substance to other anatomical areas (Figures 2, 3).

Although there are no globally accepted management guidelines, the diagnostic criteria include the histopathological findings of a biopsy, and several authors agree that it is appropriate to perform it as part of the diagnostic process.^1,10,11^ In this case, biopsies of the lesions on the face and right buttock revealed dermis with adipose tissue disaggregation, spaces of variable size surrounded by mixed inflammation and foreign-body giant cells, with a Swiss cheese appearance. Staining ruled out infection (Figure 4). These findings are consistent with the typical histological descriptions of ASIA syndrome according to Alijotas-Reig.^9^

The literature on dermatological findings in biopolymer diseases is limited, but foreign body granulomas, characterized by foreign material surrounded by histiocytes, multinucleated giant cells, and other inflammatory cells, are considered the main feature.^11,12^ Paraffin or silicone granulomas, which present a Swiss cheese morphology, such as those found in our patient, are characteristic. However, these characteristics do not allow for absolute certainty in identifying the type of material responsible, as different substances can generate similar patterns.^9,12^

Once the diagnosis is established, the patient should be treated by a multidisciplinary team.^10^ The management of these patients is complex, as it is not standardized. Furthermore, given the diversity of substances used as fillers, it is very difficult to predict their behavior, so there is no treatment that guarantees complete success.^7,8^ However, this pathology is usually approached from a rheumatological perspective, because it is considered to be related to an altered immune autoregulatory response. Specifically, a Type IV granulomatous or hypersensitivity reaction, in which the patient could benefit from management with immunomodulators and antihistamines.^1^ In this case, both types of medications were used.

Various drugs have been utilitzed to manage biopolymer disease to reduce the inflammatory response or prevent migration of the injected material. These include nonsteroidal anti-inflammatory drugs, intralesional and systemic steroids, colchicine, antibiotics, cytotoxic agents, imiquimod, etarnecept, and methotrexate combined with folic acid.^1^ In many cases, the material has spread extensively into the tissues, rendering it unresectable, necessitating the initiation of pharmacological immunomodulation,^7^ as was performed in this case.

It is important to note that management should always be based on assessing the patient's progress and adjusting the dose according to the individualized response.^1^ In this case, despite immunomodulatory management with adalimumab and leflunomide, it was decided to adjust the dose of corticosteroids and colchicine and switch the immunomodulatory therapy to methotrexate. Upon admission, treatment was initiated with hydrocortisone and colchicine, which was subsequently modified based on the patient's progress. She improved significantly after the administration of prednisone, methotrexate, and colchicine, with doses that were progressively adjusted according to her clinical behavior.

Despite the patient's improvement at the follow-up visit, some skin lesions were still evident, so it was decided to optimize management. Considering that this was a Type IV hypersensitivity reaction, it was important to supplement treatment with antihistamines, so hydroxyzine and then bilastine were added.^13^ Additionally, during follow-up, the patient was found to have elevated transaminases. This finding was considered precipitated by methotrexate, given its known hepatotoxic effects^16^). Therefore, the dose was reduced, because this helps reduce the side effects of methotrexate.^17,14^

With treatment, the patient demonstrated excellent clinical progress, including improvement of both skin lesions and joint pain. Although the use of intralesional corticosteroids was considered, this approach was ruled out because of the extent and depth of the lesions, as well as the risk of dissemination of the infiltrated material. Systemic management with immunomodulators was prioritized given the clinical severity of the inflammatory involvement of the skin and joints.

Regarding surgical intervention, referral to plastic surgery was considered during hospitalization but was postponed until clinical stabilization with pharmacological treatment was achieved, in accordance with evidence suggesting a better prognosis after acute inflammation has been controlled.^11^ According to the experience at Hospital General de México, surgical removal should ideally be performed after stabilization of the acute inflammatory process and before complications arise.^5^ In this case, following clinical stabilization, the plastic surgery team determined that the patient would benefit from biopolymer removal. The patient is currently awaiting the procedure, pending administrative clearance from the health system.

## CONCLUSIONS

Biopolymer-induced disease represents a growing diagnostic and therapeutic challenge, especially because of its potential to trigger autoimmune phenomena such as adjuvant-induced ASIA. In this case, the importance of a comprehensive clinical evaluation and a timely multidisciplinary approach are highlighted, which led to significant clinical improvement through personalized immunomodulatory treatment. Furthermore, it is noteworthy that before the development of the skin lesions and severe inflammatory reaction, the patient had been diagnosed with rheumatoid arthritis. Patients with autoimmune diseases may develop more severe or earlier manifestations after exposure to biopolymers, possibly because of an underlying immunological predisposition. This observation highlights the need for prospective studies exploring the role of preexisting autoimmunity in the severity and progression of ASIA, as well as its impact on therapeutic approaches.
